# Calcium signalling in the acinar environment of the exocrine pancreas: physiology and pathophysiology

**DOI:** 10.1113/JP275395

**Published:** 2018-03-26

**Authors:** Oleksiy Gryshchenko, Julia V. Gerasimenko, Shuang Peng, Oleg V. Gerasimenko, Ole H. Petersen

**Affiliations:** ^1^ Cardiff School of Biosciences Cardiff University Cardiff CF10 3AX UK; ^2^ Bogomoletz Institute of Physiology Kyiv 01024 Ukraine; ^3^ Department of Physiology, Medical College Jinan University Guangzhou 510632 China; ^4^ Systems Immunity Research Institute Cardiff University Cardiff CF14 4XN UK

**Keywords:** calcium signalling, exocrine pancreas, pancreatitis

## Abstract

**Key points:**

- Ca^2+^ signalling in different cell types in exocrine pancreatic lobules was monitored simultaneously and signalling responses to various stimuli were directly compared.
- Ca^2+^ signals evoked by K^+^‐induced depolarization were recorded from pancreatic nerve cells. Nerve cell stimulation evoked Ca^2+^ signals in acinar but not in stellate cells.
- Stellate cells are not electrically excitable as they, like acinar cells, did not generate Ca^2+^ signals in response to membrane depolarization.
- The responsiveness of the stellate cells to bradykinin was markedly reduced in experimental alcohol‐related acute pancreatitis, but they became sensitive to stimulation with trypsin.
- Our results provide fresh evidence for an important role of stellate cells in acute pancreatitis. They seem to be a critical element in a vicious circle promoting necrotic acinar cell death. Initial trypsin release from a few dying acinar cells generates Ca^2+^ signals in the stellate cells, which then in turn damage more acinar cells causing further trypsin liberation.

**Abstract:**

Physiological Ca^2+^ signals in pancreatic acinar cells control fluid and enzyme secretion, whereas excessive Ca^2+^ signals induced by pathological agents induce destructive processes leading to acute pancreatitis. Ca^2+^ signals in the peri‐acinar stellate cells may also play a role in the development of acute pancreatitis. In this study, we explored Ca^2+^ signalling in the different cell types in the acinar environment of the pancreatic tissue. We have, for the first time, recorded depolarization‐evoked Ca^2+^ signals in pancreatic nerves and shown that whereas acinar cells receive a functional cholinergic innervation, there is no evidence for functional innervation of the stellate cells. The stellate, like the acinar, cells are not electrically excitable as they do not generate Ca^2+^ signals in response to membrane depolarization. The principal agent evoking Ca^2+^ signals in the stellate cells is bradykinin, but in experimental alcohol‐related acute pancreatitis, these cells become much less responsive to bradykinin and then acquire sensitivity to trypsin. Our new findings have implications for our understanding of the development of acute pancreatitis and we propose a scheme in which Ca^2+^ signals in stellate cells provide an amplification loop promoting acinar cell death. Initial release of the proteases kallikrein and trypsin from dying acinar cells can, via bradykinin generation and protease‐activated receptors, induce Ca^2+^ signals in stellate cells which can then, possibly via nitric oxide generation, damage more acinar cells and thereby cause additional release of proteases, generating a vicious circle.

## Key points

- Ca^2+^ signalling in different cell types in exocrine pancreatic lobules was monitored simultaneously and signalling responses to various stimuli were directly compared.
- Ca^2+^ signals evoked by K^+^‐induced depolarization were recorded from pancreatic nerve cells. Nerve cell stimulation evoked Ca^2+^ signals in acinar but not in stellate cells.
- Stellate cells are not electrically excitable as they, like acinar cells, did not generate Ca^2+^ signals in response to membrane depolarization.
- The responsiveness of the stellate cells to bradykinin was markedly reduced in experimental alcohol‐related acute pancreatitis, but they became sensitive to stimulation with trypsin.
- Our results provide fresh evidence for an important role of stellate cells in acute pancreatitis. They seem to be a critical element in a vicious circle promoting necrotic acinar cell death. Initial trypsin release from a few dying acinar cells generates Ca^2+^ signals in the stellate cells, which then in turn damage more acinar cells causing further trypsin liberation.

## Introduction

Ca^2+^ signalling studies on isolated pancreatic acinar cells (PACs) or small acinar cell clusters have led to a detailed understanding of the mechanisms underlying the primary intracellular Ca^2+^ release elicited by physiological and pathological agents as well as the subsequent opening of store‐operated Ca^2+^ channels in the plasma membrane that accounts for the secondary Ca^2+^ entry from the extracellular solution (Petersen & Tepikin, 2008; Petersen *et al*. 2017). Physiological, short‐lasting and repetitive local Ca^2+^ signals control acinar fluid and enzyme secretion (Petersen, 1992; Petersen & Tepikin, 2008), whereas sustained global elevations of the cytosolic Ca^2+^ concentration ([Ca^2+^]_i_), elicited by pathological agents, play a key role in the development of the acinar cell damage and death leading to acute pancreatitis (AP) (Gerasimenko *et al*. 2014). Most of the work on PAC Ca^2+^ signalling has been carried out on isolated mouse cells, but the key results have been confirmed in studies on isolated human PACs (Murphy *et al*. 2008; Liang *et al*. 2017). A limited amount of work on acinar cell Ca^2+^ signalling in pancreatic segments has confirmed that the basic character of such signals, as established in isolated cell studies, is also valid in the intact pancreas (Ashby *et al*. 2003).

PACs dominate the exocrine pancreatic tissue (Bolender, 1974), but there are other important cell types. In addition to the acinar fluid secretion, there is a ductal secretion process whereby a HCO_3_^−^‐rich fluid is produced, which is important for neutralizing in the gut the acid secretion from the stomach (Hegyi & Petersen, 2013). Ca^2+^ signals in the pancreatic duct cells play an important role in the control of HCO_3_^−^ secretion, and excessive Ca^2+^ signal generation, as in the acinar cells, causes Ca^2+^ overload and toxicity (Maleth & Hegyi, 2014).

More recently, Ca^2+^ signalling and ion channels have been studied in pancreatic stellate cells (PSCs) (Fels *et al*. 2016; Ferdek *et al*. 2016; Gryshchenko *et al*. 2016; Nielsen *et al*. 2017; Storck *et al*. 2017). The role of these cells in normal physiology is unclear, but they have long been suspected of contributing to the fibrosis occurring in chronic pancreatitis as well as pancreatic cancer (Ferdek & Jakubowska, 2017; Pang *et al*. 2017). In the normal pancreas, PSCs can be observed as thin elongated structures situated at the acinar periphery, very close to the basal surface of the PACs (Gryshchenko *et al*. 2016). In spite of the close proximity of PSCs and PACs, they are not directly connected. Thus Ca^2+^ signals specifically generated in PACs are not transmitted to neighbouring PSCs and vice versa (Gryshchenko *et al*. 2016). The principal physiological agents eliciting Ca^2+^ signals in PACs are acetylcholine (ACh) and cholecystokinin (CCK), but they have no effect on PSCs (Gryshchenko *et al*. 2016). Bradykinin (BK) is the principal agent evoking Ca^2+^ signals in normal PSCs (Gryshchenko *et al*. 2016), but this peptide has no direct effect on PACs (Gryshchenko *et al*. 2016). Furthermore, PACs and PSCs possess different bile acid transporters. Whereas taurocholate and cholate elicit Ca^2+^ signals in PSCs, because they are taken up into these cells by Na^+^‐dependent transporters, these bile acids hardly evoke any Ca^2+^ signals in the PACs. On the other hand, the bile acid taurolithocholic acid sulphate (TLC‐S) evokes clear Ca^2+^ signals in PACs, but has no effect on PSCs (Ferdek *et al*. 2016).

In spite of the absence of evidence for any direct connection between neighbouring PACs and PSCs, there is indirect evidence showing that Ca^2+^ signal generation in PSCs can have profound effects on PACs. Thus the level of PAC necrosis evoked by the bile acid TLC‐S, which acts selectively on PACs, is markedly enhanced by stimulation with BK, which only acts on PSCs (Ferdek *et al*. 2016). Furthermore, the level of PAC necrosis elicited by a mixture of bile acids or by a fatty acid ethyl ester (FAEE), is markedly reduced by a BK type 2 receptor antagonist (Gryshchenko *et al*. 2016). Because Ca^2+^ signals in PSCs generate nitric oxide (NO), whereas this is not the case in PACs, it is possible that the effects of PSC Ca^2+^ signals on PACs are mediated by NO diffusing from PSCs into PACs (Jakubowska *et al*. 2016).

Early studies by Scheele and Haymovits (1978, 1980) indicated that PACs are electrically excitable, as K^+^ depolarization evoked Ca^2+^‐dependent enzyme secretion from guinea pig PACs, which could not be blocked by atropine. However, it turned out that the secretory response was due to the Ca^2+^‐dependent release of a non‐cholinergic, non‐adrenergic neurotransmitter, probably vasoactive intestinal polypeptide (VIP) and its action on the PACs (Pearson *et al*. 1981a,b). It is now well established that PACs are electrically non‐excitable, as they cannot fire action potentials, and do not possess voltage‐activated Ca^2+^ channels (Petersen, 1992). The functional innervation of PACs by parasympathetic nerves is physiologically important and has been studied in some detail (Petersen, 1992), but it is unknown whether PSCs are functionally innervated.

PSCs can undergo significant transformations and this occurs in pancreatitis (Ferdek & Jakubowska, 2017; Pang *et al*. 2017), but it is not known how this would affect Ca^2+^ signal generation in PSCs in response to various stimuli.

The aim of the study presented here was to provide a more complete description of cellular Ca^2+^ signalling events in and around the acinar units in the normal pancreas than has previously been available. Furthermore, we were interested in comparing PSC Ca^2+^ signalling properties in the pancreas from mice with experimental AP with those in the normal tissue, as any changes could have implications for our understanding of the mechanism underlying AP.

Our results demonstrate, that – in addition to observing Ca^2+^ signals in PACs and PSCs – it is possible to record Ca^2+^ signals from nerve cells in the peri‐acinar environment. However, in contrast to the clear evidence for functional innervation of the PACs, we did not observe Ca^2+^ signals in PSCs in response to nerve stimulation. Experimental AP caused major changes in PSC Ca^2+^ signalling. In alcohol‐related AP, induced by intra‐peritoneal injections of ethanol and fatty acids, there was a markedly reduced responsiveness to BK, but the PSCs were now able to generate substantial Ca^2+^ signals when stimulated by trypsin. These results provide fresh evidence for a significant role of PSCs in the destructive processes leading to AP.

## Methods

### Ethical approval

All regulated procedures carried out on animals involved in this publication were approved by Cardiff University's Animal Welfare and Ethical Review Body (AWERB), and covered by a Project Licence granted by the Home Office under the Animal (Scientific Procedures) Act, 1986. All animals were killed humanely according to the Schedule 1 protocol by cervical dislocation. Before and throughout the experiment, mice were maintained in plastic cages with corn cob bedding; tap water and commercial pelleted diet were freely provided. The mice were killed before the removal of the pancreas according to Schedule 1 of the UK Animals Act. The investigators understand the ethical principles under which *The Journal of Physiology* operates and state that this work complies with these principles.

### Induction of experimental AP

To establish AP in C57BL6/J mice (Charles River, Wilmington, MA, USA), they received two intraperitoneal injections of ethanol (1.35 g kg^−1^) and palmitoleic acid (POA) (150 mg kg^−1^), at 1 h intervals, preceded by injection of PBS, as previously described (Wen *et al*. 2015; Huang *et al*. 2017). Because it has been established that fatty acids and ethanol can react together inside cells to produce FAEEs (Criddle *et al*. 2006; Huang *et al*. 2014), we refer to this pancreatitis model as FAEE‐AP (Wen *et al*. 2015; Huang *et al*. 2017). Control mice received injections of the PBS solution alone. Humane killing was 48 h after the last injection.

### Histology

Pancreatic tissue was fixed in 4% formaldehyde and embedded in paraffin, and histological assessment was performed after haematoxylin and eosin staining of fixed pancreatic slices (4 μm thickness). Evaluation was performed on ≥10 random fields (magnification: ×200) by two blinded independent investigators grading (scale, 0–3) oedema, inflammatory cell infiltration and acinar necrosis as previously described (Van Laethem *et al*. 1996; Wen *et al*. 2015), calculating the means ± SEM (*n* = 3 mice per group).

### Lobule preparation

Pancreatic lobules were isolated from the pancreas of adult normal mice (Gryshchenko *et al*. 2016) or from mice in which AP had been induced as described above. The pancreas was rapidly dissected, transferred to a collagenase Na^+^‐Hepes‐based solution and incubated for 5–6 min at 37°C. Thereafter, the tissue was kept in a standard medium with the following composition (in mm): NaCl, 140; KCl, 4.8; Hepes (KOH), 10; MgCl_2_, 1; CaCl_2_, 1; glucose, 10; pH 7.3. In experiments where the effects of omitting extracellular Ca^2+^ were investigated, CaCl_2_ was left out of the standard solution. In experiments where the effects of membrane depolarization were investigated, the medium contained 100 mm KCl and the NaCl concentration was reduced to 44.8 mm. Pancreatic lobules were then incubated with fluorescent dye following the manufacturer's description. All experiments on normal pancreatic lobules were carried out with fresh preparations attached to the coverslip of a perfusion chamber at room temperature (∼23°C). In experiments on lobules in which the effects of exposure to fatty acids and ethanol were investigated, the lobules were exposed to a medium containing POA (20 μm) and ethanol (12 mm) for 2.5 h before starting the experiments.

The pancreas is dominated quantitatively by exocrine cells, but also contains endocrine cells, in particular insulin‐secreting β‐cells. The endocrine cells are found in the islets of Langerhans and these can be identified as dense and discrete spherical or ovoid structures sharply delineated from the surrounding more translucent exocrine tissue (Dean & Matthews, 1970). We deliberately did not focus on these structures as it was our objective to specifically study Ca^2+^ signalling events in the acinar environment.

### Ca^2+^ measurements

Pancreatic lobules were loaded with 5 μm Fluo‐4 acetoxymethyl ester (AM), for 20 min at room temperature. The tissue was transferred into a flow chamber and superfused with the Na^+^‐Hepes‐based extracellular solution as described above. Cells were visualized using a Leica SP5 MPII two‐photon confocal microscope, with an ×63 1.3 NA objective lens. Fluo‐4 was excited with a 488 nm argon laser, at 1–4% power, and emitted light was collected at 500–580 nm. Generally, a series of images was recorded at 512 × 512 pixels resolution (at the speed of 1 frame s^–1^), and analysed using Leica Confocal Software (Leica, Mannheim, Germany). Fluorescence signals were plotted as *F*/*F*_0_ (*F*_0_ is the initial level of fluorescence). In many experiments three‐dimensional recording in time have been conducted (2–3 images per time point). Statistical analysis was performed using ANOVA or Student's *t*‐test.

## Results

### General approach

**Figure 1:**
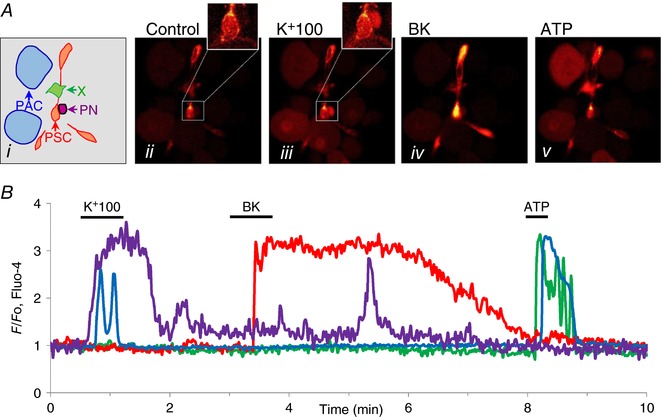
Simultaneous recordings of [Ca^2+^]_i_ changes in response to various stimuli in four different cell types in a mouse pancreatic lobule *Ai*, sketch of location of different cell types in the lobule: blue, PACs; orange/red, PSCs; purple, PN; green, unknown (X). *Aii–v*, fluorescence images in control and during stimulation with high K^+^ (100 mm), BK (1 nm) and ATP (100 μm). As also seen in the [Ca^2+^]_i_ traces shown in *B*, PN and PACs displayed rises in [Ca^2+^]_i_ in response to membrane depolarization. PSCs responded to BK and both PACs and X responded to ATP. The colours of the traces in *B* match the coloured arrows in *Ai*.

Our general aim was to simultaneously study signalling in the various cell types to be found in the acinar environment in a live pancreatic lobule preparation. Figure 1 shows an example. As previously demonstrated (Gryshchenko *et al*. 2016), PSCs take up Ca^2+^‐sensitive fluorescent probes much more avidly than PACs, so the initial assumption – looking at the fluorescence intensity levels in the resting situation (Fig. 1 *Aii*) – was that the bright cells represent PSCs. To check whether nerve cells were present and, if so, to test whether nerve stimulation could elicit Ca^2+^ signals in PACs or other cells, a solution with a high (100 mm) K^+^ concentration was introduced. As seen in Fig. 1 *Aiii*, this caused a rise in [Ca^2+^]_i_ in several relatively large cells, which must be the always quantitatively dominant PACs. Importantly, there was no rise in [Ca^2+^]_i_ in the PSCs, but in one cell – partly ‘hidden’ by a PSC – there was a large Ca^2+^ signal. This cell is most likely a neuron (PN). The apparently unprovoked short‐lasting Ca^2+^ signal in this cell occurring later in the experiment may be due to a spontaneous action potential or a short burst of action potentials. The assumption that the bright cells seen in Fig. 1 *Aii* were PSCs was confirmed when these cells became significantly brighter, indicating rises in [Ca^2+^]_i_, after stimulation with BK (1 nm) (Fig. 1 *Aiv*). Finally, the lobule was stimulated by ATP (100 μm), which caused a rise in [Ca^2+^]_i_ in the PACs and in a cell (green in the schematic diagram in Fig. 1 *Ai*) that had not reacted to high K^+^ or BK exposure. The nature of this cell is unclear and it is therefore labelled X.

**Figure 2:**
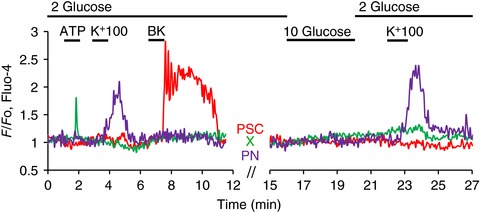
Elevating the extracellular glucose concentration from 2 to 10 mm has no effect on [Ca^2+^]_i_ in any of the peri‐acinar cells In this experiment the lobule preparation was superfused with a solution containing 2 mm glucose, which then only late in the experimental protocol was replaced by 10 mm glucose for a few minutes. As shown in the green trace, an X‐cell responded to ATP (100 μm) with a rise in [Ca^2+^]_i_, but did not respond to subsequent challenges with high K^+^ (100 mm), BK (1 nm) or 10 mm glucose. In contrast, the PSC (red trace) did not respond to ATP or high K^+^, but only to BK. The PSC also failed to respond to the stimulation with 10 mm glucose. The PN (purple trace) only responded, repeatedly, to the high‐K^+^ stimulus.

As mentioned in the Methods, the pancreas contains insulin‐secreting β‐cells, in addition to the quantitatively dominant exocrine cells. It has long been known that PACs possess insulin receptors and that insulin can affect PAC functions including Ca^2+^ signalling (Sankaran *et al*. 1981; Singh, 1985; Mankad *et al*. 2012; Samad *et al*. 2014). As described in the Methods, we did not explore Ca^2+^ signalling in or near the islets of Langerhans and it would therefore seem very unlikely that any of the cells in the acinar environment we investigated could be insulin‐secreting β‐cells or would be influenced by local insulin secretion. We nevertheless checked this by using the standard protocol for eliciting Ca^2+^ signals in β‐cells, namely by testing the effect of elevating the external glucose concentration (Dean & Matthews, 1970) from 2 to 10 mm. As seen in Fig. 2, none of the peri‐acinar cell types generated Ca^2+^ signals in response to glucose stimulation (see also further details in the sections below on PNs and X‐cells).

### Ca^2+^ signals in pancreatic nerve cells (PNs)

**Figure 3:**
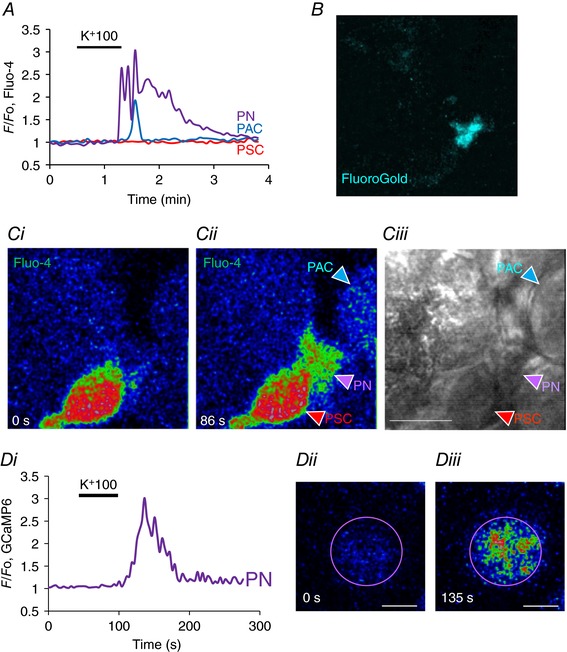
K^+^ depolarization evokes Ca^2+^ signals in labelled pancreatic neurons *A*, K^+^ depolarization evoked a rise in [Ca^2+^]_i_ in a FluoroGold labelled PN (*B*) as well as in a PAC. *Ci–ii*, fluorescence images before (0 s) and during the high K^+^ challenge (86 s) showing the evoked rise in [Ca^2+^]_i_ in the PN as well as the PAC, but with no change in the PSC. *Ciii*, transmitted light image of the field, also showing location of the different cells. Length of horizontal bar corresponds to 10 μm. *D*, ultrasensitive protein calcium sensor GCaMP6 was expressed in mouse pancreas by intravenous injection of adeno‐associated virus AAV9.Syn.GCaMP6s (Penn Vector Core at University of Pennsylvania) targeted to neurons. K^+^ depolarization evoked a significant rise in [Ca^2+^]_i_ (*Di*). As seen in *Dii*, at rest the PN had relatively low fluorescence (0 s) but this increased by a factor of three after depolarization with high‐K^+^ solution (*Diii*, 135 s). Length of horizontal bars in *Dii* and *Diii* corresponds to 5 μm. No other cells in the field of view displayed any changes in fluorescence intensity. In this experiment the only fluorescent probe present was GCaMP6.

It is clear from experiments of the type shown in Fig. 1 that there are cells other than PACs that respond to depolarization with Ca^2+^ signals. One possibility is that they are PNs and we therefore tested this hypothesis. The fluorescent dye FluoroGold has been demonstrated to undergo retrograde axonal transport and stains nerve cells (Naumann *et al*. 2000). Figure 3 *A*–*C* shows the results from an experiment (*n* = 3) in which a FluoroGold‐labelled cell (Fig. 3 *B*) responded to membrane depolarization, elicited by a high‐K^+^ (100 mm) solution, with an increase in [Ca^2+^]_i_ (Fig. 3 *A*). A non‐FluoroGold‐labelled PAC also produced a rise in [Ca^2+^]_i_, presumably due to the action of ACh released from nerve endings (see below), whereas a PSC failed to respond (Fig. 3). We also undertook experiments in which the ultra‐sensitive Ca^2+^ sensor GCaMP6 was expressed in mice by intravenous injection of adeno‐associated virus AAV9.Syn.GCaMP6s targeted to neurons (Chen *et al*. 2013). As seen in Fig. 3 *Di*–*iii*, a short‐lasting high‐K^+^ stimulation caused a substantial transient increase in [Ca^2+^]_i_ (*n* = 7).

**Figure 4:**
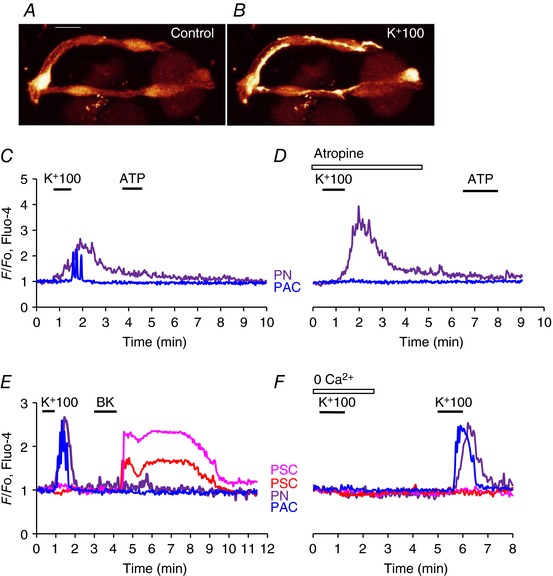
K^+^ depolarization evokes Ca^2+^ signals in PNs that depend on extracellular Ca^2+^ but, unlike the signals in PACs, are not blocked by atropine *A* and *B*, fluorescence images illustrating that PNs sometimes have elongated parts that seem very closely linked to PSCs. The images show Fluo‐4 fluorescence before (*A*) and during high‐K^+^ stimulation (*B*). Length of horizontal bar in *A* corresponds to 10 μm. *C*, high‐K^+^ induced Ca^2+^ signals in a PN and a PAC, but subsequent stimulation with ATP did not elicit any responses. *D*, in the presence of atropine (10 μm), high K^+^ still evoked a Ca^2+^ signal in the PN, but no longer in the PAC. *E*, high K^+^ elicited Ca^2+^ signals in PN and PAC, but not in two PSCs in which subsequently Ca^2+^ signals were observed in response to BK stimulation. BK did not elicit Ca^2+^ signals in PN and PAC. *F*, in the absence of external Ca^2+^, K^+^ depolarization failed to elicit Ca^2+^ signals in PN and PAC. After reintroduction of the Ca^2+^‐containing external solution, high K^+^ was again able to evoke Ca^2+^ signals in PN and PAC, but not in two PSCs.

Many of the pancreatic cells that have neuron‐like properties are located close to PSCs (see Fig. 1). In several cases (*n* = 14) we could observe Ca^2+^ signal propagation in PNs along the bodies and elongated parts of PSCs as a pathway through the lobules (Fig. 4 *A*, *B*).

**Figure 5:**
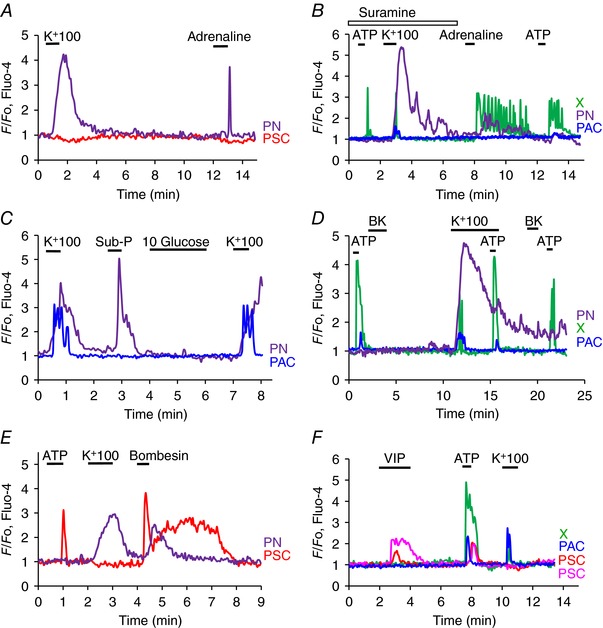
The effects of various neurotransmitters on [Ca^2+^]_i_ in the different cell types found in the lobules *A*, the PN, but not the PSC, produces a Ca^2+^ signal in response to high‐K^+^ stimulation and subsequently generates a Ca^2+^ signal in response to stimulation with adrenaline (20 μm). *B*, in the presence of the purinergic receptor antagonist suramin, high‐K^+^ stimulation evokes a normal Ca^2+^ signal in the PN and a very short‐lasting signal in the X‐cell. The effect of ATP (100 μm) on the X‐cell in the presence of the purinergic antagonist is very short‐lasting compared to the effect of the same concentration of ATP later in the same cell after wash‐out of suramin. Adrenaline (20 μm) evoked a train of Ca^2+^ spikes in the X‐cell, but in this experiment only had a questionable effect on the PN. *C*, high K^+^ elicited Ca^2+^ signals in PAC and PN. Substance P (10 μm) evoked a Ca^2+^ signal in the PN, but not in the PAC. Glucose (10 mm) failed to evoke Ca^2+^ signals in both the PN and the PAC (in these experiments the standard solution contained only 2 mm glucose). *D*, ATP repeatedly evoked Ca^2+^ signals in X‐cell and small signals in PAC, but not in PN. High‐K^+^ stimulation evoked large Ca^2+^ signal in PN, but only a short‐lasting signal in the X‐cell. The ATP‐elicited Ca^2+^ signal in the X‐cell was not diminished during the period of high‐K^+^ depolarization. BK did not evoke any effects in these three cells. *E*, ATP and bombesin (1 μm) evoked Ca^2+^ signals in a PSC and bombesin also elicited a Ca^2+^ signal in a PN that responded to high‐K^+^ stimulation. *F*, VIP (100 nm) evoked Ca^2+^ signals in two PSCs, but neither in a PAC nor in a X‐cell, whereas ATP produced Ca^2+^ signals in all the cells (X, PAC and PSC). High K^+^ evoked Ca^2+^ signals in the X‐cell and the PAC, but not in the PSCs.

The rise in [Ca^2+^]_i_ in PNs, elicited by K^+^‐induced depolarization (Figs 1, 2, 3, 4, 5) could potentially be influenced by release of neurotransmitters from nerve cells not visualized in the segment under investigation and we therefore tested possible effects of various neurotransmitters (Figs 4 and 5). Figure 4 *C* and *D* shows examples of Ca^2+^ signals in a PN and a PAC generated by exposure to a high‐K^+^ solution. The PAC signal, as expected, was clearly not mediated by depolarization of the acinar cell membrane as it was abolished by atropine (*n* = 12), in agreement with the well‐established cholinergic innervation of PACs (Petersen, 1992), whereas the Ca^2+^ signal in the PN could still be observed in the presence of this muscarinic antagonist (*n* = 17). ATP did not have any effects on PNs (*n* > 100; Fig. 4 *C*, *D*), whereas this agent could, in several cases, produce Ca^2+^ signals in PACs and PSCs (Fig. 1), although not in the case shown in Fig. 4 *C* and *D*. Ca^2+^ signals induced by the high‐K^+^ solution in both PACs and PNs were reversibly abolished by removal of external Ca^2+^ (*n* = 4) (Fig. 4 *E*, *F*). Ca^2+^ signals elicited by a high‐K^+^ solution in PNs were not inhibited by the non‐selective purinergic antagonist suramin (*n* = 5; Fig. 5 *B*).

Several neurotransmitters elicited Ca^2+^ signals in PNs. Adrenaline (20 μm) evoked signals in 21 out of 35 PNs tested (Fig. 5 *A*). This response was mediated by α‐ rather than β‐receptors as the β‐adrenergic agonist isoprenaline had no effect (*n* = 10) whereas the α‐receptor agonists cirazoline (50 μm) and UK 14.304 (50 μm) could mimic the effect of adrenaline (*n* = 8). Ca^2+^ signals in PNs were also elicited by Substance P (10 μm) in 4 out of 7 neurons (Fig. 5 *C*). Bombesin, which is known to elicit Ca^2+^ signals in PACs by interaction with receptors that are distinct from the CCK receptors (Deschodt‐Lanckman *et al*. 1976; Iwatsuki & Petersen, 1978), evoked Ca^2+^ signals in 7 out of 8 PNs (Fig. 5 *E*). On the other hand, PNs did not respond to BK (*n* > 100; Fig. 5 *D*) or VIP (*n* = 4).

In the hypothalamus there are neurons responsive to glucose (Burdakov *et al*. 2005) and in the pancreas the insulin‐secreting β‐cells have long been known to depolarize and fire action potentials when challenged with glucose above a certain threshold concentration (Dean & Matthews, 1970; Dean *et al*. 1975; Atwater *et al*. 1978). We therefore tested whether the PNs in our preparation would be sensitive to changes in the extracellular glucose concentration. In these experiments the glucose concentration in the fluid surrounding the lobules was kept low (2 mm) for a prolonged period (15–30 min) before exposure to 10 mm glucose. As seen in Fig. 5 *C*, a PN in which a high‐K^+^ solution, as well as Substance P, elicited Ca^2+^ signals failed to respond to stimulation with 10 mm glucose (*n* = 13).

### PSCs are not electrically excitable but respond to some neurotransmitters

As previously described, PSCs consistently generate Ca^2+^ signals when challenged with BK (Fig. 1; Ferdek *et al*. 2016; Gryshchenko *et al*. 2016), but it is not known whether they are functionally innervated. We never observed Ca^2+^ signals in PSCs when lobules were exposed to high‐K^+^ solutions (*n* > 100). Figure 4 *E* shows the result of an experiment in which a high‐K^+^ solution elicited Ca^2+^ signals in both a PN and a PAC without evoking a response from two PSCs, which both subsequently generated Ca^2+^ signals when stimulated by BK.

As previously shown (Gryshchenko *et al*. 2016), PSCs could (Fig. 5 *EF*), but did not always (Fig. 1), generate Ca^2+^ signals in response to ATP (100 μm) stimulation. Bombesin (1 μm) elicited Ca^2+^ signals in some PSCs (*n* = 8 out of 21 cells tested; Fig. 5 *E*) and VIP (100 nm) could evoke Ca^2+^ signals in 37 out of the 78 PSCs tested (Fig. 5 *F*).

### X‐cells

As shown in Fig. 1 there is an unknown (X) cell type that generates a substantial Ca^2+^ signal in response to ATP stimulation (*n *> 100). In these ATP‐sensitive X‐cells, high‐K^+^ stimulation could in many, but not all, cases evoke short‐lasting Ca^2+^ signals (Fig. 5 *D*), but once these cells had been challenged with a high‐K^+^ pulse, they needed a long recovery time (>30 min) before they could respond again (*n* = 9). In these cells, adrenaline (20 μm) could evoke Ca^2+^ signals (Fig. 5 *B*, *n* = 14 out of 33 cells tested), but X‐cells never responded to BK (*n* > 100) (Fig. 5 *D*). We also tested whether the X‐cells were glucose‐sensitive. We used the same protocol as for the similar experiments testing glucose sensitivity in PNs (Fig. 5 *C*) (low – 2 mm – basal glucose concentration and then a test pulse of 10 mm glucose). In five experiments, X‐cells that responded to ATP stimulation with Ca^2+^ signals failed to generate any increase in [Ca^2+^]_i_ in response to 10 mm glucose (Fig. 2).

### Alcohol‐related AP changes the responsiveness of PSCs

As described in the Introduction, it is known that PSCs undergo morphological and functional changes during pancreatitis (Ferdek & Jakubowska, 2017; Pang *et al*. 2017) and we were therefore interested in exploring whether their responsiveness to BK would also change as a result of this transformation. We investigated this in two different ways. In one type of experiment, we induced changes in the pancreatic lobule preparation, similar to those seen in AP, by exposing the tissue to a mixture of ethanol and POA, which is known to generate palmitoleic acid ethyl ester (POAEE) inside PACs (Laposata & Lange, 1986; Criddle *et al*. 2006; Huang et al 2014; 2017). In the second type of experiment, we induced AP in mice *in vivo*, by injections of ethanol and POA, and then removed the pancreas to investigate Ca^2+^ signalling properties in the lobule preparation.

**Figure 6:**
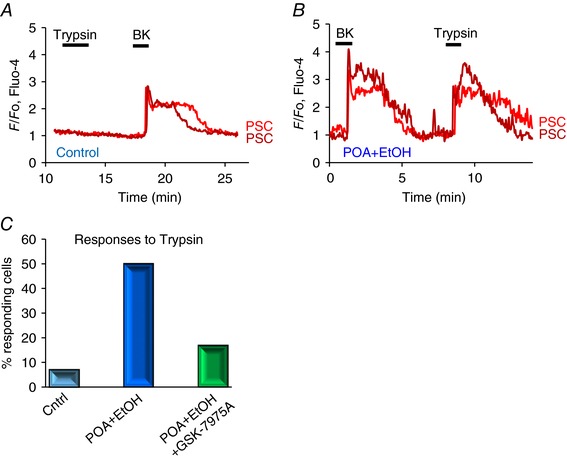
Exposure of pancreatic lobules to a mixture of POA and ethanol induces PSC responsiveness to trypsin In *A* and *B*, the effects of trypsin (50 nm) and BK (1 nm) on [Ca^2+^]_i_ in PSCs in a control lobule are compared with those in lobules that had been exposed to a mixture of POA (20 μm) and ethanol (12 mm) for 2.5 h. In control PSCs, trypsin (50 nm) only evoked a Ca^2+^ signal in 2 cells out of 28 tested, and not in the case shown in *A*, whereas the same concentration of trypsin evoked a clear Ca^2+^ signal in the PSC in a lobule that had been treated with POA and ethanol (*n* = 14 out of 28 cells tested). *C*, summary of the results of the experiments illustrated in *A* and *B*, showing the marked increase in the percentage of PSCs responding to trypsin with Ca^2+^ signals after POA/ethanol exposure. In the presence of the CRAC channel inhibitor GSK‐7975A (20 μm), the percentage of PSCs responding to trypsin in the POA/ethanol groups was markedly reduced (*n* = 12 of 71 cells tested).

Figure 6 summarizes the results from the *in vitro* series of experiments. In the control lobules (no POA/ethanol) we confirmed that PSCs respond to BK (1 nm) stimulation by generating substantial Ca^2+^ signals and also confirmed the previously reported result that trypsin does not elicit Ca^2+^ signals (Gryshchenko *et al*. 2016). However, following exposure to POA/ethanol, the cells produced substantial Ca^2+^ signals in response to a concentration (50 nm) of trypsin that had failed to elicit signals in the control preparations (Fig. 6). As seen in Fig. 6, the effect of trypsin was acute and reversible and could therefore not be a consequence of cell death induced by digestion, but must be a receptor‐mediated (protease‐activated receptor) effect. Many PSCs may well have died during the exposure to POA/ethanol, but the cells from which [Ca^2+^]_i_ recordings were made were still viable, as seen by their ability to bring [Ca^2+^]_i_ back to the control level after a short exposure to trypsin (Fig. 6 *B*). The proportion of PSCs responding to trypsin in the AP lobules was markedly reduced by including the CRAC channel inhibitor GSK‐7975A in the POA/ethanol solution used to generate AP (Fig. 6 *C*). Because CRAC channel inhibition has been shown to reduce store‐operated Ca^2+^ influx in both PACs and PSCs, this supports the idea previously proposed (Ferdek *et al*. 2016; Gryshchenko *et al*. 2016) that excessive Ca^2+^ signal generation in PACs as well as PSCs play a central role in the development of AP.

**Figure 7:**
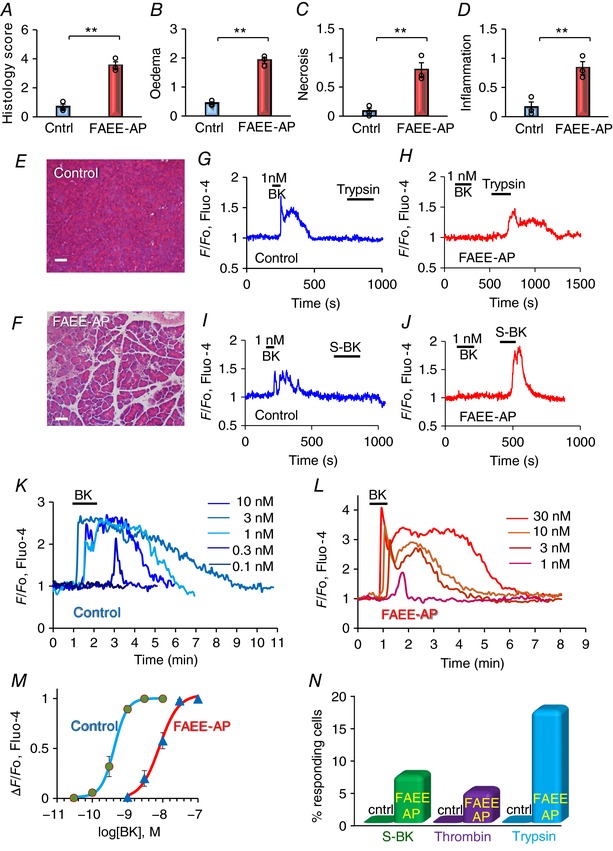
Functional changes in PSCs due to alcohol‐induced AP (POA/ethanol *in vivo* mouse model – FAEE‐AP) *A–F*, the successful induction of AP was investigated by histological assessments of fixed pancreatic slices. Comparisons were made between pancreatic slices from control mice and FAAE‐AP mice. Overall histology score (*A*), degree of oedema (*B*), extent of necrosis (*C*) and degree of inflammation (*D*) were recorded (^**^ *P *< 0.01). Representative images of pancreatic histology sections from control (*E*) and FAEE‐AP (*F*) mice are also shown (bars: 50 μm). In each case the number of independent experiments (from different mice) = 3 (but in each experiment >20 sections were examined; typically ∼1000–2000 cells in each experimental group). *G–J*, representative [Ca^2+^]_i_ traces from PCSs in lobules from a control mouse (*G*, *I*) and a mouse with AP (*H*, *J*). In the control lobules (*G*, *I*), BK (1 nm) consistently evoked Ca^2+^ signals, whereas trypsin (10 nm) and the selective BK 1 receptor agonist Sar‐[DPhe8]‐des‐Arg8‐bradykinin (S‐BK) (1 μm) failed to do so. In the lobules isolated from mice with FAEE‐AP, BK (1 nm) failed to elicit Ca^2+^ signals, but trypsin (10 nm) and S‐BK (1 μm) were able to elicit such signals. *K–M*, quantitative evaluation of the change in PSC sensitivity to BK following induction of FAEE‐AP. *K* shows traces of BK‐elicited [Ca^2+^]_i_ changes, all from one and the same PSC, in a control lobule, whereas *L* shows the results, from one and the same PSC, in a lobule from an FAAE‐AP mouse. The data from all experiments are summarized by the concentration–response curves in *M* (*n* = 6–10 for each point). *N*, comparisons of the responsiveness of PSCs to S‐BK (1 μm), thrombin (5 mU ml^–1^) and trypsin (10 nm) in control and FAEE‐AP lobules.

In the *in vivo* experiments, we verified that AP had been induced by evaluating pancreatic histology sections, comparing tissue from control mice with those that had been injected with POA/ethanol. Figure 7 *A*–*F* summarizes these data. It can be seen that the overall histology score, the degree of oedema, the level of acinar necrosis and the extent of immune cell invasion were all markedly increased in the pancreatic tissue from the mice that had been injected with POA/ethanol as compared to the normal tissue. As seen in Fig. 7 *H*, *J* and *L* and *M*, the PSCs in the AP mice, in contrast to the control mice (Fig. 7 *G*, *I*, *K* and *M*), hardly responded to 1 nm BK, but in a number of PSCs Ca^2+^ signals in response to trypsin (10 nm) were observed (*n* = 8 out of 38 cells tested, Fig. 7 *H* and *N*). Control PSCs did not respond to trypsin Fig. 7 *G*), as also previously reported (Gryshchenko *et al*. 2016). Similar to the effects of trypsin on PSCs in lobules exposed *in vitro* to POA/ethanol mixtures (Fig. 6 *B*), the actions of this enzyme in this series of experiments (Fig. 7 *H* and *N*) were also acute and reversible, indicating a receptor‐mediated (protease‐activated receptor) effect rather than a consequence of cell damage. Although many PSCs may have been destroyed by the actions of POA/ethanol *in vivo*, clearly those responding to trypsin were intact. In a few cases (*n* = 3 out of 59 cells tested), thrombin (5 mU ml^−1^), another protease, evoked Ca^2+^ signals in PSCs in lobules from FAEE‐AP mice, whereas this was not observed in control tissue, as also previously reported (Gryshchenko *et al*. 2016). Because Ca^2+^ signals in normal PSCs evoked by BK is due to activation of type 2 BK receptors (Gryshchenko *et al*. 2016), the desensitization to BK seen in AP (Fig. 7 *K*–*M*) would appear to represent a specific desensitization of the type 2 receptors. This is supported by the finding that although PSCs in the FAEE‐AP tissue failed to respond to a BK concentration (1 nm) that elicited a maximal Ca^2+^ signal in the control tissue, some PSCs from FAEE‐AP lobules could produce Ca^2+^ signals when stimulated with a high concentration (1 μm) of a BK agonist specific for type 1 BK receptors (S‐BK) (Fig. 7 *J* and *N*; 8 cells out of 101 tested). In contrast, S‐BK only evoked a Ca^2+^ signal in one PSC out of 118 tested in lobules from control mice. Figure 7 *N* summarizes the results of the experiments comparing the responsiveness of PSCs to S‐BK, thrombin and trypsin in control and FAEE‐AP.

We evaluated quantitatively the reduced responsiveness of the PSCs to BK in AP by comparing concentration–response curves for BK‐elicited Ca^2+^ signal generation in control and AP (Fig. 7 *K*–*M*). The results show that the concentration–response curve was shifted markedly to the right in AP, as compared to the control values. Thus a BK concentration of 1 nm, which evokes a near‐maximal Ca^2+^ signal in control PSCs (Gryshchenko *et al*. 2016; Fig. 7 *K* and *M*, *n* = 11), hardly evoked any change in [Ca^2+^]_i_ in the PSCs from the AP lobules (Fig. 7 *L* and *M*, *n* = 12).

## Discussion

In this study of Ca^2+^ signalling in the peri‐acinar environment of the exocrine pancreas, we have for the first time been able to record Ca^2+^ signals from PNs, and demonstrated that PSCs are not electrically excitable and, in contrast to the PACs, do not appear to be functionally innervated. We have also discovered a hitherto unknown cell type (X) which is very responsive to stimulation with ATP (Figs 1, 2 and 5). Further investigations will be needed to establish the character and function of this cell type.

**Figure 8:**
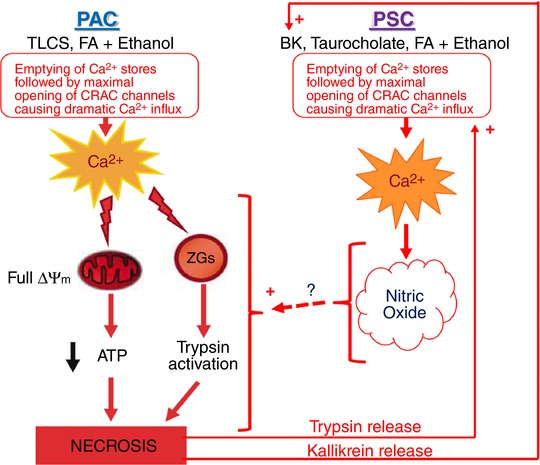
Schematic diagram illustrating how the PSCs may participate in a vicious circle amplifying necrotic PAC death in AP In the PACs the initial damage leading to AP may be caused by a combination of fatty acids (FA) and ethanol or by certain bile acids (for example, TLC‐S). These agents generate excessive Ca^2+^ signals in the PACs causing mitochondrial depolarization and therefore reduced mitochondrial ATP production (Gerasimenko *et al*. 2014). The excessive Ca^2+^ signals also cause intracellular trypsin activation. Necrosis in at least a proportion of PACs follows, releasing activated proteases, including trypsin and kallikrein into the interstitial fluid. Kallikrein catalyses the formation of BK from bradykininogen and BK in turn acts on PSCs to generate Ca^2+^ signals. Trypsin acts in the same way. These actions would amplify the direct actions of certain bile acids (e.g. taurocholate) and possibly fatty acids and ethanol on the PSCs. In these cells, Ca^2+^ signals activate the enzyme NO synthase, thereby producing NO and this gas may diffuse into neighbouring PACs and there, by mechanisms not yet understood, promote the necrotic process. This will then lead to additional protease release, further stimulating the PSCs, generating a vicious circle.

Our new results show that experimental induction of AP causes a major change in the responsiveness of PSCs to BK. In the normal pancreas, PSCs generate small, but clear, Ca^2+^ signals in response to stimulation with 0.1 nm BK, a concentration only slightly above the resting plasma concentration of this agent (Blais *et al*. 1999; Hirata *et al*. 2002), and near‐maximal Ca^2+^ signals at a BK concentration of 1 nm. In contrast, PSCs in lobules isolated from the pancreas in mice with FAEE‐AP need more than 1 nm BK to produce clear signals and require a BK concentration of 30 nm to produce maximal Ca^2+^ signals. The BK concentration–response curve is thus shifted markedly to the right by induction of AP (Fig. 7 *M*). This desensitization is probably due to BK liberation from bradykininogen, as a result of kallikrein release from PACs undergoing necrosis (Schachter 1969; Orlov & Belyakov, 1978; Griesbacher *et al*. 2003), causing a prolonged exposure of the PSCs to an elevated tissue level of BK (Griesbacher *et al*. 2003). These data in conjunction with our recent demonstration that a BK receptor antagonist markedly reduced the extent of PAC necrosis induced in lobules by exposure to FAEEs or bile acids (Gryshchenko *et al*. 2016) and our finding that bile‐induced damage to PACs can be markedly enhanced by BK stimulation of PSCs (Ferdek *et al*. 2016) suggest that PSCs may be critically involved in a vicious circle promoting PAC necrosis. Figure 8 shows a schematic model in which initial damage to PACs would lead to release of activated enzymes, including kallikrein, into the interstitial fluid, causing an increase in the BK concentration, which would then generate Ca^2+^ signals in PSCs. These Ca^2+^ signals – via NO formation in PSCs and with NO diffusing into adjacent PACs (Jakubowska *et al*. 2016) – would contribute to further damage of PACs, which in turn would cause further release of kallikrein leading to a further increase in the BK level and thereby further stimulation of PSCs acting to promote PAC necrosis. We have shown that pharmacological inhibition of NO synthase provides remarkable protection against necrosis (Jakubowska *et al*. 2016), but the mechanism by which NO exerts this effect is unknown.

The BK‐induced Ca^2+^ signals in PSCs are mediated by BK type 2 receptors (Gryshchenko *et al*. 2016) and in normal PSCs an analogue of BK that selectively interacts with type 1 receptors does not elicit Ca^2+^ signals. This changes after induction of AP (Fig. 7), as PSCs can now produce Ca^2+^ signals in response to a high concentration of the BK type 1 receptor agonist S‐BK (Fig. 7 *N*). Given that the concentration of the type 1 agonist that is required to evoke a signal is very high, this may not in itself have any functional significance, but the reduced sensitivity to type 2 receptor activation and increase in sensitivity to type 1 activation is a general feature of inflammatory diseases (Petho & Reeh, 2012).

The decrease in PSC sensitivity to BK, induced by ethanol and POA, takes time to develop. Thus the exposure of pancreatic lobules to POA/ethanol for 150 min is insufficient (Fig. 6 *AB*), whereas induction of FAEE‐AP over 48 h *in vivo* produces a clear reduction in responsiveness to BK (Fig. 7). The relatively short period (150 min) of exposure of pancreatic tissue to POA/ethanol is, however, sufficient for a proportion of PSCs to become sensitive to trypsin, an enzyme that has no effect on Ca^2+^ signalling in normal PSCs (Fig. 6). In the *in vivo* FAEE‐AP model there was also induction of sensitivity to trypsin (Fig. 7). These results may indicate the existence of a further necrotic amplification loop in which initial damage to PACs, induced – for example – by FAEEs or a bile acid, causes release of activated trypsin from dying PACs, which in turn activates PSCs to produce Ca^2+^ signals, generating NO that may diffuse into neighbouring PACs, thereby causing further cell death (Jakubowska *et al*. 2016) and therefore further liberation of trypsin and other proteases (Fig. 8). Thrombin may also have a role in AP (Andersson *et al*. 2010) and this enzyme sometimes induced Ca^2+^ signals in PSCs from AP lobules, but not in normal PSCs (Fig. 7). Thrombin would also act via activation of protease‐activated receptors (Coughlin, 2000). The responsiveness to trypsin, and to some extent perhaps also to thrombin, highlights the importance of the protease‐activated receptors in FAEE‐AP. We have recently provided evidence for the importance of these receptors in drug‐induced AP (Peng *et al*. 2016).

Although PSCs have not traditionally been thought to play a role in AP, our new results strengthen the case for such an involvement that began to emerge from our previous investigations of PSCs (Ferdek *et al*. 2016; Gryshchenko *et al*. 2016; Jakubowska *et al*. 2016). Specifically, our new data indicate initial roles for BK, followed by trypsin, generating Ca^2+^ signals in PSCs (Fig. 8) and provide fresh evidence in favour of the propositions made many years ago, but largely ignored, that inhibition of BK receptors could have benefits in the treatment of AP (Griesbacher *et al*. 1993; Hirata *et al*. 2002).

We have previously shown that CRAC channel inhibition markedly reduces the prolonged [Ca^2+^]_i_ elevation due to the store‐operated Ca^2+^ entry into the PSCs that follows the initial BK‐elicited intracellular Ca^2+^ release (Gryshchenko *et al*. 2016). Since then it has been shown that PSCs possess Ca^2+^‐activated K^+^ channels (Storck *et al*. 2017) and it is therefore likely that initial Ca^2+^ release from intracellular stores would activate such channels, promoting store‐operated Ca^2+^ entry due to the more favourable electrochemical gradient provided by the hyperpolarized plasma membrane. Inhibition of excessive Ca^2+^ signal generation in PACs and PSCs by partial blockade of CRAC channels is a promising therapeutic avenue in many inflammatory diseases (Parekh, 2010; Di Capite *et al*. 2011) including AP (Gerasimenko *et al*. 2013, 2014; Wen *et al*. 2015). Our new data (Fig. 6 *C*), showing that CRAC channel inhibition largely prevents the increased responsiveness of PSCs to trypsin that occurs in AP‐like conditions, provides fresh evidence in favour of CRAC channel inhibition as a potentially attractive treatment for AP.

## Additional information

### Competing interests

The authors declare no competing interests.

### Author contributions

All experiments were carried out in the School of Biosciences at Cardiff University. All authors jointly conceived the project. OG and JVG carried out the experiments with help from SP, and these were then also analysed by OVG. JVG created the figures and OHP wrote the paper with significant intellectual input from all the other authors. All authors have approved the final version of the mansucript.

### Funding

The work was supported by grants from the Medical Research Council (UK) (MR/J002771/1 and G19/22/2).

**Translational perspective**

Our new data indicate that the pancreatic stellate cells play an important role in acute pancreatitis. They are key amplifying elements in a process resulting in necrotic acinar cell death. Initial release of proteases–including trypsin and kallikrein–from a small proportion of dying acinar cells generates Ca^2+^ signals in the stellate cells which then, probably via formation of the diffusible gas nitric oxide in these cells, causes more acinar necrosis which, via release of proteases from the further damaged acinar cells, causes additional stellate cell stimulation, thereby generating a vicious circle. These findings have potential therapeutic implications, as they indicate that interventions that would break this vicious circle could be helpful in the treatment of acute pancreatitis, a disease for which there is currently no authorized rational therapy. The key elements in the vicious circle promoting acinar necrosis would appear to be the actions of bradykinin, generated by the action of kallikrein, and trypsin on specific stellate cell receptors. These actions cause rises in the cytosolic Ca^2+^ concentration in the stellate cells, which activate the Ca^2+^‐sensitive enzyme nitric oxide synthase, producing the very diffusible nitric oxide, which in this situation appears to be toxic for the acinar cells. There are clear pharmacological interventions that could prove effective. Bradykinin receptor antagonists, antagonists of protease‐activated receptors and inhibitors of nitric oxide synthase could all be helpful. Given that excessive Ca^2+^ signal generation in both stellate and acinar cells are critical elements, our previous proposal of treating acute pancreatitis with inhibitors of store‐operated Ca^2+^ entry via the so‐called CRAC channels, which has received further support from our new results, remains valid. However, it may well turn out that combination therapy with Ca^2+^ channel inhibitors and, for example, bradykinin receptor antagonists, could be particularly helpful and would allow relatively low doses of these agents to be used, thereby minimizing potential side effects.
